# Leishmaniose tegumentar americana: análise epidemiológica e série temporal, Minas Gerais, 2008-2024

**DOI:** 10.1590/S2237-96222026v35e20251212.pt

**Published:** 2026-11-09

**Authors:** Marcony Ian Monção Pereira, Pedro Vitor de Souza Silva, Sara Luiza Ramos Ribeiro, Sabrina Gonçalves Paixão Souza, Camila Lorrany Rodrigues Ferreira, Vanessa de Andrade Royo, Dario Alves de Oliveira, Afrânio Farias de Melo, Murilo Malveira Brandao, Elytania Veiga Menezes

**Affiliations:** 1 Universidade Estadual de Montes Claros, Departamento de Biologia Geral, Montes Claros, MG, Brasil; 2 Universidade Estadual de Montes Claros, Programa de Pós-Graduação em Biotecnologia, Montes Claros, MG, Brasil

**Keywords:** Leishmaniose Cutânea, Epidemiologia, Vigilância em Saúde Pública, Doenças Negligenciadas, Epidemiologia Descritiva, Leishmaniasis Cutánea, Epidemiología, Vigilancia en Salud Pública, Enfermedades Desatendidas, Epidemiología Descriptiva

## Abstract

**Objetivo:**

Analisar o perfil epidemiológico e a tendência temporal da leishmaniose tegumentar americana na macrorregião Norte de Minas Gerais entre anos de 2008 e 2024.

**Métodos:**

Realizou-se um estudo descritivo com base em dados de notificações oficiais do Sistema de Informação de Agravos de Notificação. Foram calculadas as frequências das características dos pacientes e as taxas de incidência (número de casos por 100 mil habitantes). A análise de tendência foi realizada pelo teste de Mann-Kendall, adotando-se nível de significância de 5%.

**Resultados:**

No período analisado, foram confirmados 7.355 casos da doença na região. A análise estatística indicou uma tendência de estabilidade na transmissão, com uma taxa média anual de 26,27 casos por 100 mil habitantes, variando de 25,80 em 2008 a 21,94 em 2024. A maioria das infecções ocorreu no próprio município de residência dos pacientes (91,0%). O perfil predominante foi de homens (59,8%), pessoas que se autodeclaram pardas (61,4%) e adultos na faixa de 15 a 59 anos (64,3%). A forma cutânea da doença foi a mais comum (93,5%), e a taxa de cura atingiu 79,6% dos casos registrados.

**Conclusão:**

A leishmaniose tegumentar americana mantém um padrão endêmico estável no Norte de Minas Gerais. O predomínio de casos locais e o impacto em grupos vulneráveis reforçam a necessidade de intensificar as ações de vigilância e o diagnóstico precoce para mitigar o agravo na região.

## Resumen

**Objetivo:**

Analizar el perfil epidemiológico y la tendencia temporal de la leishmaniasis cutánea americana en la macrorregión Norte de Minas Gerais entre 2008 y 2024.

**Métodos:**

Se realizó un estudio descriptivo con base en datos oficiales de notificación del Sistema de Información de Enfermedades de Declaración Obligatoria. Se calcularon las frecuencias de las características de los pacientes y las tasas de incidencia (número de casos por 100.000 habitantes). El análisis de tendencias se realizó mediante la prueba de Mann-Kendall, con un nivel de significancia del 5%.

**Resultados:**

Durante el período analizado, se confirmaron 7.355 casos de la enfermedad en la región. El análisis estadístico indicó una tendencia de estabilidad en la transmisión, con una tasa anual promedio de 26,27 casos por 100.000 habitantes, que osciló entre 25,80 en 2008 y 21,94 en 2024. La mayoría de las infecciones ocurrieron en el municipio de residencia de los pacientes (91,0%). El perfil predominante fue el de hombres (59,8%), personas que se autoidentificaron como mestizas (61,4%) y adultos de 15 a 59 años (64,3%). La forma cutánea de la enfermedad fue la más común (93,5%), y la tasa de curación alcanzó el 79,6% de los casos registrados.

**Conclusión:**

La leishmaniasis cutánea americana mantiene un patrón endémico estable en el norte de Minas Gerais. El predominio de casos locales y el impacto en los grupos vulnerables refuerzan la necesidad de intensificar las acciones de vigilancia y el diagnóstico precoz para mitigar la enfermedad en la región.

## Introdução

A leishmaniose tegumentar americana é uma doença infecciosa e parasitária causada por protozoários do gênero *Leishmania*. A transmissão aos humanos ocorre por meio da picada de fêmeas de flebotomíneos infectadas. Com ampla dispersão no território brasiliero, essa enfermidade é classificada pela Organização Mundial da Saúde como uma doença tropical negligenciada [3]. Devido à sua morbidade, impõe-se como um obstáculo persistente às políticas de saúde pública no Brasil [3,4]. Suas manifestações clínicas, que incluem lesões cutâneas e mucosas, comprometem severamente a qualidade de vida, podendo causar incapacidade funcional e estigmatização social dos indivíduos acometidos [5]. 

No Brasil, a leishmaniose tegumentar americana tem caráter endêmico e está associada a múltiplos fatores socioambientais. A expansão urbana desordenada, aliada à modificação de ecossistemas naturais e às desigualdades no acesso aos serviços de saúde, favorece a persistência da transmissão [6,7]. Nesse cenário, regiões de maior vulnerabilidade registram taxas de incidência elevadas, resultantes da complexa interação entre o vetor, o hospedeiro humano e as condições ambientais que sustentam o ciclo biológico [8]. 

Em Minas Gerais, a doença apresenta registros frequentes em diversas regiões, com destaque para o Norte do estado, que concentra importantes áreas de risco [6,9,10]. Nessas localidades, a ocorrência de casos demonstra flutuações temporais influenciadas pela sazonalidade do vetor e pela efetividade das ações de controle [6,9]. 

Apesar da relevância da leishmaniose tegumentar americana, persistem lacunas sobre sua dinâmica no Norte de Minas Gerais, onde particularidades socioambientais sustentam a transmissão. Este estudo utiliza uma série temporal extensa para identificar padrões de persistência e subsidiar intervenções estratégicas em saúde pública. Assim, objetivou-se analisar o perfil epidemiológico e a tendência temporal da doença na referida macrorregião, entre 2008 e 2024.

## Métodos

### Delineamento do estudo

Trata-se de um estudo epidemiológico descritivo, de base populacional, baseado em dados secundários de notificação compulsória. O período de análise compreendeu os anos de 2008 a 2024.

### Contexto

O estudo abrangeu a macrorregião Norte de Minas Gerais (86 municípios; 128.454 km^2^). A região tem 1.636.540 habitantes, conforme o Censo de 2022, e predominância de clima semiárido.

### Participantes

Foram incluídos todos os registros confirmados de leishmaniose tegumentar na macrorregião Norte de Minas Gerais no período definido. Por tratar-se de base de dados agregada e anonimizada, não houve contato direto com indivíduos.

### Variáveis

Foram analisadas variáveis sociodemográficas e clínicas. As sociodemográficas incluíram: sexo, raça/cor, região de residência e faixa etária agrupada por ciclos de vida (0-14, 15-59 e ≥60 anos). As variáveis clínicas compreenderam: forma clínica, tipo de entrada, critério de confirmação, ocorrência autóctone e evolução do caso. 

### Fonte de dados

Os dados foram obtidos do Sistema de Informação de Agravos de Notificação, processado pelo Departamento de Informática do Sistema Único de Saúde [11]. A extração foi realizada em 16 de março de 2026, via ferramenta de Tabulação de Dados.

### Viés

O uso de dados secundários apresenta risco de viés de registro e subnotificação.

### Tamanho do estudo

A amostra foi composta da totalidade de casos notificados para o recorte espacial e temporal definido, totalizando os dados disponíveis.

### Método estatístico

As frequências e taxas de incidência (por 100 mil habitantes) foram calculadas no Microsoft Excel. A tendência temporal foi analisada no software R (v. 4.5.1) pelo teste de Mann-Kendall (pacote Kendall), com nível de significância de 5% (p-valor<0,05). Utilizou-se esse teste por ser um método não paramétrico robusto a valores discrepantes e indicado para detectar tendências monotônicas, sem exigir a normalidade dos dados. Optou-se pela análise agregada para garantir o poder estatístico e evitar a instabilidade decorrente do baixo número de casos em estratos geográficos menores.

## Resultados

No período de 2008 a 2024, foram registrados 7.355 casos de leishmaniose tegumentar americana no Norte de Minas Gerais. O perfil predominante foi de indivíduos do sexo masculino (59,8%), autodeclarados pardos (61,4%) e na faixa etária de 15 a 59 anos (64,3%) (Tabela 1). Quanto aos aspectos clínicos, a forma cutânea foi a mais frequente (93,5%), com a maioria dos casos sendo classificada como caso novo (92,3%). A confirmação laboratorial ocorreu em 88,5% dos registros, e a taxa de cura foi de 79,6%.

**Tabela 1:** Características sociodemográficas, clínicas e epidemiológicas de casos de leishmaniose tegumentar americana, macrorregião Norte de Minas Gerais, 2008-2024 (n=7.355)

*Variáveis*	*n (%)*
*Regiões de Saúde*	
Montes Claros	1.750 (23,8)
Januária	1.318 (17,9)
Brasília de Minas	1.186 (16,1)
Taiobeiras	715 (9,7)
Manga	679 (9,2)
São Francisco	324 (4,4)
Pirapora	318 (4,3)
Salinas	306 (4,2)
Coração de Jesus	244 (3,3)
Francisco Sá	206 (2,8)
Bocaiúva	196 (2,7)
Janaúba/Monte Azul	113 (1,5)
Total	7.355 (100,0)
*Sexo*	
Masculino	4400 (59,8)
Feminino	2955 (40,2)
*Raça/cor*	
Parda	4.514 (61,4)
Branca	1.194 (16,2)
Preta	547 (7,4)
Indígena	458 (6,2)
Amarela	84 (1,1)
Ignorado/em branco	558 (7,6)
*Faixa etária (anos)*	
0-14	1.046 (14,2)
15-59	4.729 (64,3)
≥60	1.580 (21,5)
*Tipo de entrada*	
Caso novo	6.788 (92,3)
Reicidiva	443 (6,0)
Ignorado/em branco	124 (1,7)
*Forma clínica*	
Cutânea	6.875 (93,5)
Mucosa	480 (6,5)
*Autóctone do município de residência*	
Sim	6.693 (91,0)
Indeterminado	418 (5,7)
Não	244 (3,3)
*Critério de confirmação*	
Clínico-laboratorial	6.509 (88,5)
Clínico-epidemiológico	846 (11,5)
*Evolução dos casos*	
Cura	5.852 (79,6)
Mudança de diagnóstico	87 (1,2)
Abandono	66 (0,9)
Transferência	47 (0,6)
Óbito por outra causa	54 (0,7)
Óbito por leishmaniose tegumentar americana	8 (0,1)
Ignorado/em branco	1.241 (16,9)

A distribuição espacial dos casos (Figura 1) revela que a transmissão ocorreu em toda a macrorregião, com maior concentração de notificações nas regiões de saúde de Montes Claros (23,8%), Januária (17,9%) e Brasília de Minas (16,1%), Juntas, somam mais da metade dos registros da região.

A análise da série temporal (Figura 2) evidenciou oscilações na taxa de incidência anual. O pico de transmissão ocorreu em 2020 (41,06/100 mil habitantes) e o menor índice em 2016 (14,34/100 mil habitantes). Apesar dessas flutuações, o teste de Mann-Kendall indicou uma estabilidade temporal na incidência de leishmaniose tegumentar americana (p-valor 0,773), sem tendência significativa de aumento ou redução no período estudado.

**Figura 1:**
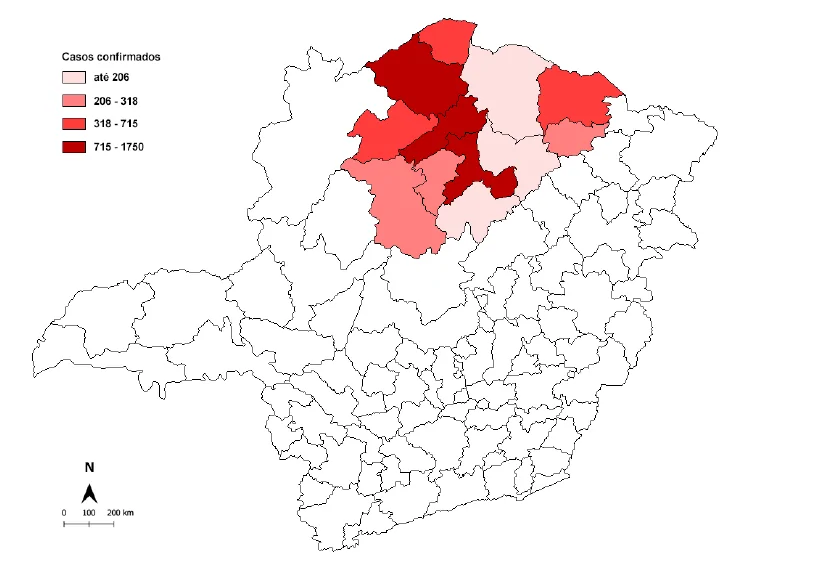
Distribuição espacial de casos confirmados de leishmaniose tegumentar americana por regiões de saúde, macrorregião Norte de Minas Gerais, 2008-2024 (n=7.355)

**Figura 2:**
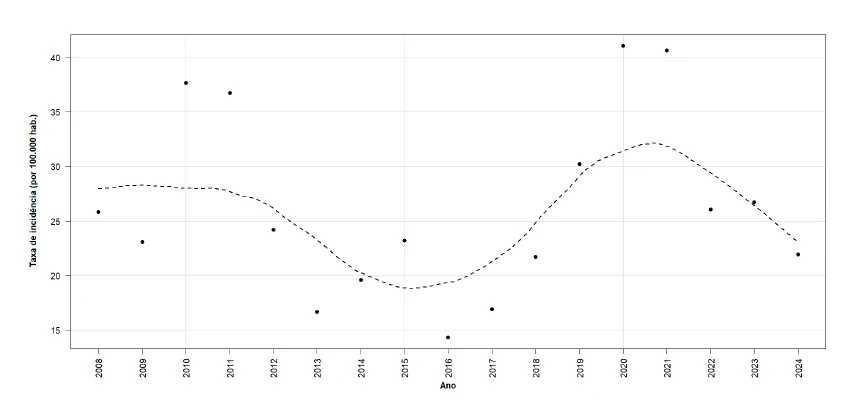
Evolução temporal da taxa de incidência de leishmaniose tegumentar americana por 100 mil habitantes na macrorregião Norte de Minas Gerais, 2008-2024

Nota: Os pontos representam as taxas anuais registradas, enquanto a linha tracejada indica a tendência suavizada da incidência no período.

## Discussão

A leishmaniose tegumentar americana no Norte de Minas Gerais é ocorre de forma constante. Diferente do observado no Brasil e nas Américas, onde os casos estão em declínio, nessa região a transmissão continua ativa. O perfil das pessoas atingidas revela que a doença afeta diversos segmentos da população, com uma incidência considerável entre os idosos. Isso indica que o ciclo de transmissão está muito bem adaptado às condições locais, sobrevivendo às formas atuais de combate e mantendo um peso preocupante de sofrimento para a população. 

Este estudo apresenta limitações inerentes ao uso de dados secundários do Sistema de Informação de Agravos de Notificação, sujeitos a subnotificações [12,13]. A incompletude de variáveis como escolaridade e renda limitou a caracterização do perfil socioeconômico dos pacientes [1,12,13]. Ademais, a presença de registros ignorados ou em branco comprometeu a qualidade da análise epidemiológica, o que pode ter mascarado a real magnitude do agravo e dificultado o planejamento de ações de vigilância mais eficientes. Por fim, a ausência de análise municipal impediu identificar padrões locais que detalhariam a dinâmica da transmissão regional.

A estabilidade na incidência encontrada no Norte de Minas Gerais merece destaque, uma vez que o Brasil e as Américas registraram queda geral nos casos nas últimas décadas [12-14]. Enquanto no país os números abaixaram, a região Sudeste, puxada por Minas Gerais, apresentou um comportamento diferente, com aumento ou manutenção dos riscos desde 2014 [1]. O menor número de casos em 2016 reflete um ano atípico em todo o país [12,13]. Por outro lado, o pico em 2020 sugere que, mesmo com a pandemia, a busca por ajuda médica não parou, possivelmente porque os efeitos colaterais da doença geram estigmatização dos indivíduos [1].

A alta autoctonia (91%) evidencia que a doença está urbanizada, em razão do crescimento desordenado das áreas urbanas sobre as matas nativas [6,15]. Em locais como Montes Claros, a destruição de florestas para a construção de novos condomínios aproximou as pessoas de insetos que transmitem a doença, a exemplo do *Nyssomyia intermedia* e do *Nyssomyia whitmani*, que conseguem viver próximos a esses ambientes [2,6]. A presença de animais de criação e de roedores perto das moradias ajuda a manter esse ciclo, pois servem de alimento para os insetos transmissores [1,6,15].

Clinicamente, a presença da forma mucosa em 6,5% dos casos é um sinal de alerta, pois esse valor é maior do que a média das Américas, que é de 4,5% [14]. A forma mucosa associa-se ao diagnóstico tardio ou falha terapêutica [13,16]. Esse dado sugere dificuldades no acesso à saúde por parte de populações e evidencia a circulação da *Leishmania braziliensis*, que é o agente etiológico comum na região [13,16]. A vulnerabilidade social se manifesta no perfil epidemiológico, visto que a maioria dos atingidos são pessoas pardas (61,4%) e homens em faixa etária de trabalho ativo [12,16]. Além disso, o grande número de idosos afetados representa um desafio, pois o tratamento padrão pode ser muito forte e causar efeitos colaterais graves nesse grupo [1,12,16]. 

Embora este estudo tenha se limitado à análise de tendência temporal, a transmissão ativa na macrorregião corrobora os inquéritos espaciais de 2021 [6], que demonstraram aglomerados significativos de alto risco (Moran I = 0,385) em 40 setores censitários rurais e periféricos de Montes Claros, e de 2018 [9], que definiram o Norte de Minas como um circuito espacial prioritário, responsável por 33,1% das notificações do estado entre 2007 e 2009. No presente estudo, a complexidade desse cenário é reforçada por indicadores operacionais, como a proporção de autoctonia indeterminada de 5,7% e a vulnerabilidade de populações pardas (61,4%) e indígenas (6,2%), sinalizando desafios diretos na vigilância. Tais grupos enfrentam distâncias geográficas acentuadas e entraves que dificultam o diagnóstico precoce e o manejo clínico em áreas remotas [13,16]. Assim, a estabilidade da doença no Norte de Minas reflete não apenas fatores biológicos mas a persistência de desigualdades estruturais que demandam políticas públicas sensíveis às especificidades territoriais dessas populações.

As estratégias de controle na região parecem estar estagnadas, com foco em tratar indivíduos doentes [13]. Para mudar esse cenário, as políticas públicas precisam ir além dos hospitais e envolver o planejamento das cidades e o cuidado com o meio ambiente [17]. Sem investir em melhorias nas moradias, no saneamento e na educação sobre a doença, o Norte de Minas continuará enfrentando dificuldades para reduzir os casos [5,16,17]. 

A leishmaniose no Norte de Minas Gerais configura-se como uma endemia estacionária que desafia as estratégias tradicionais de controle. Para reverter esse cenário, são fundamentais intervenções intersetoriais e sustentadas fundamentadas na abordagem de saúde única. Isso requer a articulação entre o diagnóstico precoce, o manejo clínico adequado, o controle vetorial estratégico e melhorias no planejamento urbano e ambiental, visando reduzir de forma efetiva a vulnerabilidade das populações regionais.

## Data Availability

O banco de dados e os códigos de análise utilizados na pesquisa estão disponíveis em: https://doi.org/10.5281/zenodo.19288854 [18]
