# Supplementary material for: Leishmaniose tegumentar americana: análise epidemiológica e série temporal, Minas Gerais, 2008-2024
Source: Epidemiol Serv Saude. 2026 Nov 9;35:e20251212. [Article in Portuguese] doi: 10.1590/S2237-96222026v35e20251212.pt (PMC13750719; doi:10.1590/S2237-96222026v35e20251212.pt)
Supplement: Translation

# Translation: American tegumentary leishmaniasis: epidemiological analysis and time series, Minas Gerais, Brazil, 2008-2024

**DOI:** 10.1590/S2237-96222026v35e20251212.en

**Authors:** Marcony Ian Monção Pereira, Pedro Vitor de Souza Silva, Sara Luiza Ramos Ribeiro, Sabrina Gonçalves Paixão Souza, Camila Lorrany Rodrigues Ferreira, Vanessa de Andrade Royo, Dario Alves de Oliveira, Afrânio Farias de Melo, Murilo Malveira Brandao, Elytania Veiga Menezes

**Affiliations:** 1 Universidade Estadual de Montes Claros, Departamento de Biologia Geral, Montes Claros, MG, Brazil; 2 Universidade Estadual de Montes Claros, Programa de Pós-Graduação em Biotecnologia, Montes Claros, MG, Brazil

**Keywords:** Leishmaniasis, Cutaneous, Epidemiology, Public Health Surveillance, Neglected Diseases, Epidemiology, Descriptive

## Abstract

**Objective:**

To analyze the epidemiological profile and temporal trend of American cutaneous leishmaniasis in the Northern macroregion of Minas Gerais state, Brazil, between 2008 and 2024.

**Methods:**

A descriptive study was conducted based on official case report data held on the Notifiable Health Conditions Information System. Frequencies of patient characteristics and incidence rates (number of cases per 100,000 inhabitants) were calculated. Trend analysis was performed using the Mann-Kendall test, adopting a 5% significance level.

**Results:**

During the period analyzed, 7,355 cases of the disease were confirmed in the region. Statistical analysis indicated a stable transmission trend, with an average annual rate of 26.27 cases per 100,000 inhabitants, ranging from 25.80 in 2008 to 21.94 in 2024. Most infections occurred in the patients’ own municipality of residence (91.0%). The predominant profile comprised males (59.8%), people who self-identified as mixed-race (61.4%) and adults aged 15 to 59 years (64.3%). The cutaneous form of the disease was the most common (93.5%), and the cure rate reached 79.6% of recorded cases.

**Conclusion:**

American tegumentary leishmaniasis maintains a stable endemic pattern in Northern Minas Gerais. The predominance of local cases and the impact on vulnerable groups highlight the need to intensify surveillance actions and early diagnosis in order to mitigate the disease in the region.

## Introduction

American tegumentary leishmaniasis is an infectious and parasitic disease caused by protozoa of the *Leishmania* genus. Transmission to humans occurs by being bitten by infected female sandflies. Widely dispersed in Brazilian territory, this disease is classified by the World Health Organization as a neglected tropical disease [3]. Due to its morbidity, it poses a persistent obstacle to public health policies in Brazil [3,4]. Its clinical manifestations, which include cutaneous and mucosal lesions, severely compromise quality of life, potentially causing functional disability and social stigmatization of affected individuals [5].

In Brazil, American tegumentary leishmaniasis is endemic and associated with multiple socio-environmental factors. Unplanned urban expansion, coupled with the modification of natural ecosystems and inequalities in access to health services, favors the persistence of transmission [6,7]. In this scenario, regions of greater vulnerability record high incidence rates, resulting from the complex interaction between the vector, the human host and the environmental conditions that sustain the biological cycle [8].

In Minas Gerais state, the disease is frequently reported in several regions, particularly in the north of the state, which concentrates important risk areas [6,9,10]. In these locations, occurrence of cases shows temporal fluctuations influenced by the seasonality of the vector and the effectiveness of control actions [6,9].

Despite the relevance of American tegumentary leishmaniasis, gaps remain regarding its dynamics in Northern Minas Gerais, where specific socio-environmental factors support its transmission. This study uses an extensive time series to identify patterns of persistence and to support strategic public health interventions. As such, the objective of the study was to analyze the epidemiological profile and temporal trend of the disease in the aforementioned macro-region, between 2008 and 2024.

## Methods

### Design

This is a descriptive, population-based epidemiological study based on secondary data derived from mandatory case reporting. The analysis period was from 2008 to 2024.

### Setting

The study covered the northern macro-region of Minas Gerais state (86 municipalities; 128,454 km^2^). The region has 1,636,540 inhabitants, according to the 2022 Census, and a predominantly semi-arid climate.

### Participants

All confirmed cases of tegumentary leishmaniasis in the northern macro-region of Minas Gerais during the defined period were included. Given that an aggregated and anonymized database was used, there was no direct contact with individuals.

### Variables

Sociodemographic and clinical variables were analyzed. Sociodemographic variables included: sex, race/color, region of residence, and age grouped by life cycles (0-14, 15-59, and ≥60 years). Clinical variables included: clinical form, type of admission, confirmation criteria, autochthonous occurrence and case evolution. 

### Data source

The data were obtained from the Notifiable Health Conditions Information System, processed by the Brazilian Unified Health System Information Technology Department [11]. Data extraction was performed on March 16, 2026, using the Data Tabulation tool.

### Bias

There is a risk of reporting bias and underreporting due to the use of secondary data.

### Study size

The sample consisted of all cases reported for the spatial and temporal scope defined, totaling the available data.

### Statistical method

Frequencies and incidence rates (per 100,000 inhabitants) were calculated using Microsoft Excel. Temporal trends were analyzed using R software (v. 4.5.1) via the Mann-Kendall test (Kendall package), with a 5% significance level (p-value<0.05). This test was used because it is a non-parametric method that is robust in relation to outliers and suitable for detecting monotonic trends, without requiring data normality. Aggregate analysis was chosen to ensure statistical power and avoid instability resulting from the low number of cases in smaller geographic strata.

## Results

Between 2008 and 2024, 7,355 cases of American tegumentary leishmaniasis were recorded in Northern Minas Gerais. The predominant profile was comprised of male individuals (59.8%), those of self-declared mixed race (61.4%), and those in the 15 to 59 age range (64.3%) (Table 1). Regarding clinical aspects, the cutaneous form was the most frequent (93.5%), with the majority of cases classified as new cases (92.3%). Laboratory confirmation occurred in 88.5% of the records, and the cure rate was 79.6%.

**Table 1:** Sociodemographic, clinical and epidemiological characteristics of American tegumentary leishmaniasis cases, Northern macro-region of Minas Gerais state, 2008-2024 (n=7,355)

*Variables*	*n (%)*
*Health Regions*	
Montes Claros	1,750 (23.8)
Januária	1,318 (17.9)
Brasília de Minas	1,186 (16.1)
Taiobeiras	715 (9.7)
Manga	679 (9.2)
São Francisco	324 (4.4)
Pirapora	318 (4.3)
Salinas	306 (4.2)
Coração de Jesus	244 (3.3)
Francisco Sá	206 (2.8)
Bocaiúva	196 (2.7)
Janaúba/Monte Azul	113 (1.5)
Total	7,355 (100.0)
*Sex*	
Male	4,400 (59.8)
Female	2,955 (40.2)
*Race/color*	
Mixed-race	4,514 (61.4)
White	1,194 (16.2)
Black	547 (7.4)
Indigenous	458 (6.2)
Asian	84 (1.1)
Unknown/Left blank	558 (7.6)
*Age group (years)*	
0-14	1,046 (14.2)
15-59	4,729 (64.3)
≥60	1,580 (21.5)
*Type of admission*	
New case	6,788 (92.3)
Recurrence	443 (6.0)
Unknown/Left blank	124 (1.7)
*Forma clínica*	
Cutaneous	6,875 (93.5)
Mucosal	480 (6.5)
*Autochthonous to the municipality of residence*	
Yes	6,693 (91.0)
Undetermined	418 (5.7)
No	244 (3.3)
*Confirmation criterion*	
Clinical/laboratory	6,509 (88.5)
Clinica/epidemiological	846 (11.5)
*Case progression*	
Cure	5,852 (79.6)
Change of diagnosis	87 (1.2)
Treatment drop-out	66 (0.9)
Transfer	47 (0.6)
Death due to other cause	54 (0.7)
Death due to American tegumentary leishmaniasis	8 (0.1)
Unknown/Left blank	1,241 (16.9)

The spatial distribution of cases (Figure 1) reveals that transmission occurred throughout the macro-region, with a higher concentration of notifications in the Montes Claros (23.8%), Januária (17.9%), and Brasília de Minas (16.1%) health regions. Together, these regions account for more than half of the region’s recorded cases.

The time series analysis (Figure 2) showed fluctuations in the annual incidence rate. Peak transmission occurred in 2020 (41.06/100,000 inhabitants), while the lowest rate was in 2016 (14.34/100,000 inhabitants). Despite these fluctuations, the Mann-Kendall test indicated temporal stability in American tegumentary leishmaniasis incidence (p-value 0.773), with no significant rising or falling trend during the study period.

**Figure 1:** Spatial distribution of confirmed American tegumentary leishmaniasis cases by health regions, Northern macro-region of Minas Gerais state, 2008-2024 (n=7,355)

**Figure 2:** Temporal evolution of the American tuegumentary leishmaniasis incidence rate per 100,000 inhabitants in the Northern macroregion of Minas Gerais state, 2008-2024

Note: The dots represent the recorded annual rates, while the dashed line indicates the smoothed trend of incidence over the period.

## Discussion

American tegumentary leishmaniasis occurs consistently in Northern Minas Gerais. Unlike what is observed in Brazil as a whole and the Americas, where cases are falling, transmission remains active in this region. The profile of those affected reveals that the disease affects various segments of the population, with considerable incidence among the elderly. This indicates that the transmission cycle is very well adapted to local conditions, surviving current control methods and maintaining a worrying burden of suffering for the population.

This study has limitations inherent to the use of secondary data taken from the Notifiable Health Conditions Information System, which are subject to underreporting [12,13]. The incompleteness of variables such as education and income limited the characterization of the socioeconomic profile of patients [1,12,13]. Furthermore, missing or blank records compromised the quality of the epidemiological analysis, which may have masked the true magnitude of the disease and hindered the planning of more efficient surveillance actions. Finally, absence of municipal analysis prevented identification of local patterns that could detail the dynamics of regional transmission.

The incidence stability found in Northern Minas Gerais stands out, since Brazil and the Americas have recorded an overall decrease in cases in recent decades [12-14]. While the numbers decreased in Brazil as a whole, the Southeast region of the country, led by Minas Gerais state, showed a different behavior, with risks increasing or remaining stable since 2014 [1]. The lower number of cases in 2016 reflects an atypical year throughout Brazil [12,13]. On the other hand, the peak in 2020 suggests that, even with the pandemic, people did not stop seeking medical help, possibly because the side effects of the disease result in affected individuals being stigmatized [1].

The high autochthonous rate (91%) shows that the disease is urbanized, due to the uncontrolled growth of urban areas over native forests [6,15]. In places like Montes Claros, deforestation in order to provide sites for building new residential condominiums has brought people closer to insects that transmit the disease, such as *Nyssomyia intermedia* and *Nyssomyia whitmani*, which have the ability to live close to these environments [2,6]. The presence of farm animals and rodents near dwellings helps maintain this cycle, as they serve as food sources for the transmitting insects [1,6,15].

Clinically, the presence of the mucosal form in 6.5% of cases is a warning sign, as this value is higher than the average for the Americas, which is 4.5% [14]. The mucosal form is associated with late diagnosis or treatment failure [13,16]. This data suggests difficulties in access to healthcare for populations and highlights the circulation of *Leishmania braziliensis*, which is the common etiological agent in the region [13,16]. Social vulnerability is manifested in the epidemiological profile, since the majority of those affected are of mixed-race (61.4%) and men in the active working age range [12,16]. In addition, the large number of elderly people affected represents a challenge, as standard treatment can be very strong and cause serious side effects in this group [1,12,16].

Although this study was limited to temporal trend analysis, active transmission in the macroregion corroborates the spatial surveys conducted in 2021 [6], which demonstrated significant high-risk clusters (Moran’s I = 0.385) in 40 rural and peripheral census tracts in Montes Claros, and the survey conducted in of 2018 [9], which defined Northern Minas Gerais as a priority spatial circuit, responsible for 33.1% of the state’s reported cases between 2007 and 2009. In the present study, the complexity of this scenario is emphasized by operational indicators, such as 5.7% of autochthonous cases being undetermined and the vulnerability of mixed-race (61.4%) and Indigenous (6.2%) populations, signaling direct challenges in surveillance. These groups face significant geographical distances and obstacles that hinder early diagnosis and clinical management in remote areas [13,16]. Thus, the stability of the disease in Northern Minas Gerais reflects not only biological factors but also the persistence of structural inequalities that demand public policies sensitive to the territorial specificities of these populations.

Control strategies in the region appear to be stagnant, focusing on treating sick individuals [13]. In order to change this scenario, public policies need to go beyond hospitals and involve city planning and environmental care [17]. Without investing in housing improvements, sanitation and education about the disease, Northern Minas Gerais will continue to face difficulties in reducing cases [5,16,17].

Leishmaniasis in Northern Minas Gerais is a stationary endemic disease that challenges traditional control strategies. In order to reverse this scenario, intersectoral and sustained interventions based on a One Health approach are fundamental. This requires coordination between early diagnosis, appropriate clinical management, strategic vector control, and improvements in urban and environmental planning, aiming to effectively reduce the vulnerability of regional populations.

## Data Availability

The database and analysis codes used in this research are available at https://doi.org/10.5281/zenodo.19288854 [18]
